# EXPOSURE OF THE SWISS POPULATION BY RADIODIAGNOSTICS: 2013 REVIEW

**DOI:** 10.1093/rpd/ncv462

**Published:** 2016-06-07

**Authors:** Régis Le Coultre, Julie Bize, Mélanie Champendal, David Wittwer, Nick Ryckx, Abbas Aroua, Philipp Trueb, Francis R. Verdun

**Affiliations:** 1 University of Health Sciences (HESAV), University of Applied Sciences and Arts Western Switzerland (HES-SO), Av. de Beaumont 21, Lausanne CH – 1011, Switzerland; 2 Institute of Radiation Physics (IRA), University Hospital, CHUV, Rue du Grand-Pré 1, Lausanne CH – 1007, Switzerland; 3 Radiation Protection Division, Swiss Federal Office of Public Health, Schwarzenburgstrasse 165, Bern CH – 3003, Switzerland

## Abstract

In 2013, a nationwide investigation was conducted in Switzerland to establish the population's exposure from medical X rays. A hybrid approach was used combining the Raddose database accessible on-line by the participating practices and the Swiss medical tariffication system for hospitals. This study revealed that the average annual number of examinations is 1.2 per inhabitant, and the associated annual effective dose is 1.4 mSv. It also showed that computed tomography is the most irradiating modality and that it delivers 70 % of the total dose. The annual effective dose per inhabitant registered a 17 % increase in 5 y and is comparable with what was recently reported in neighbouring countries.

## INTRODUCTION

Medical exposure to X rays represents the population's main source of man-made irradiation. The worldwide annual effective dose per inhabitant is ∼3.1 mSv^(1)^. Diagnostic radiology represents only 20 % of this but it accounts for >94 % of the man-made exposure. In Switzerland, the annual effective dose per inhabitant in 2013 was 5.6 mSv^(2)^. Diagnostic radiology represented 21 % of the total but >92 % of the man-made irradiation. This is why surveying the population's exposure from medical X rays is recommended and regularly performed as a useful tool in radiation protection, both worldwide^(1)^ and in Europe^(3, 4)^.

Switzerland started surveying the exposure of its population from medical X rays back in the late 1950s and the exposure of the Swiss population is reviewed periodically, every decade with a full re-evaluation survey addressing all health care providers and every 5 y with an updating survey covering a small stratified sample of health care providers. The last full re-evaluation surveys concerned the 1998 and 2008 data^(4–10)^ and the last updating survey concerned the 2003 data^(11)^. These surveys provided a significant amount of information on the frequency of the X-ray examinations performed in Switzerland and the associated radiation doses as well as the main trends in diagnostic and interventional radiology. They revealed, for example, the sharp increase in the use of computed tomography.

The aim of this survey was to establish the population's exposure from medical X rays in 2013.

## MATERIALS AND METHODS

A hybrid method was used in this investigation for establishing the frequencies of X-ray examinations, combining two complementary methodologies. For small medical and dental practices and radiology institutes, the Raddose on-line database, developed for the 2008 full survey^(2)^, was used. For hospitals and clinics, the Swiss medical tariffication system (TARMED) explored previously in a pilot study^(12)^ was used.

### Raddose database

A dedicated website (raddose.ch) was developed to host a database accessible on-line. The participants received their own username and password, granting them access to this platform. They were encouraged to fill in a form with reference categories of examinations, based on the Dose-Data-Med methodology^(3)^.

### TARMED system

The system of medical tariffication Switzerland, named TARMED (http://www.tarmedsuisse.ch), consists of nearly 5000 positions and includes almost all medical and paramedical services provided in medical practices and in hospitals for outpatients. A number of tariff points are attributed to each service depending on the time required, the degree of difficulty and the equipment required. Additionally, TARMED distinguishes between medical and technical services. The data that feed the system of medical tariffication was collected in order to allow for the use of context information: date of the procedure, the permanent patient identifier (anonymized), age and sex of the patient, etc. In order to analyse the enormous amount of data provided by the TARMED system, the METAXA computer code (METAdata eXtraction an Analysis) (http://www.ingenierie-sante.ch) was used.

### Extrapolation

Six categories of health care providers were considered: hospitals and clinics, radiology institutes, general practitioners (GP), chiropractors, dentists using conventional radiography only and dentists equipped with a cone beam computed tomography facility.

For this survey, the authors contacted 603 medical and dental practices and radiology institutes and requested to provide their data on-line through the Raddose platform. This represents an average sample of 8 % of the total number. The average response rate was 28 %. The detailed sampling data, including the national sampling figures used to project the number of examinations at the national level, are given in Table 1.

**Table 1.** Sampling of small practices.

Practices and institutes	Total	Contacted	Respondents	Response rate (%)	National sampling (%)
Chiropractors	116	110	31	28.2	26.7
GP	3715	200	56	28.0	1.5
Dentists without cone beam CT	3129	100	26	26.0	0.8
Dentists with cone beam CT	323	83	22	26.5	6.8
Radiology institutes	118	110	31	28.2	26.3

It was often difficult to receive access to the TARMED data from hospitals and clinics. After several reminders, only 30 % of the total 2013 data were obtained. Data from all university hospitals, 38 large hospitals and 7 private clinics were collected. This data set was used to establish the age and sex distributions of the X-ray examinations but was not judged sufficient for determining national frequencies of examinations.

To estimate the dose at the national level, the authors decided to use one particular region of Switzerland (Vaud Canton, counting for 9.2 % of the Swiss population) as a representative sample, where ∼100 % of the data could be obtained. This region being representative of Swiss practices, including large and small medical structures in cities and remote areas, the authors considered first that it was responsible for 9.2 % of the total number of examinations (fraction of the whole population). Because the authors also had access to the total number of medical consultations performed in all the Cantons of Switzerland, they noticed that their sample (Vaud Canton) was responsible for 8.8 % of the national medical consultations. Despite the small difference between these numbers, they decided to divide the number of examinations by 0.088 rather than 0.092 to get the national figures. This assumes that the ratio of the number of radiological examinations to that of medical consultations is the same all over Switzerland—a hypothesis considered reasonable by the Swiss Society of Radiology.

### Dose data

The dose data used in this study for radiography, fluoroscopy and dental radiology was derived from the various national dose surveys carried out between 2008 and 2013. These values are fully compatible with the ones used for the French national survey^(13)^. For computed tomography, the authors checked whether the values used in the French national survey^(13)^ were compatible with Swiss practices by comparing them with the data collected during 1 y (2014) at the Lausanne University Hospital by the DoseWatch system (GE Healthcare) for 2014.

## RESULTS AND DISCUSSION

The main results of the survey are given in Table 2, which presents the average annual number of examinations per 1000 population, the average dose per examination and the average annual effective dose per inhabitant, for the various radiological modalities. Considering all types of examinations, the average annual number of examinations was found to be equal to 1.2 per inhabitant, and the associated annual effective dose was estimated to 1.4 mSv. In comparison with the 2008 results, a slight decrease in the frequencies of both medical and dental X rays was observed. On the other hand, the authors found a significant increase in interventional procedures guided by fluoroscopy.

**Table 2.** Distribution of the annual number of examinations per thousand population, dose per examination and the average per inhabitant effective dose over the various radiological modalities.

Radiological modality	Number of examinations/1000 inhabitants	Frequencies (%)	Dose (mSv)	Dose (mSv)/1000 inhabitants	Contribution to the collective dose (%)
Radiography	473	38.83	0.32	151.44	10.67
Mammography (diagnostic)	20	1.66	0.36	7.30	0.51
Mammography (screening)	11	0.93	0.36	4.06	0.29
Computed tomography	117	9.61	8.54	1000.21	70.44
Dental radiology (conventional)	572	46.91	0.02	11.44	0.81
Dental radiology (cone beam CT)	6	0.45	0.20	1.10	0.08
Conventional fluoroscopy	7	0.61	8.00	59.09	4.16
Interventional fluoroscopy for diagnostic purposes:					
Coronary angiographies	6	0.47	14.00	79.59	5.61
Other angiographies	2	0.17	8.00	16.98	1.20
Interventional fluoroscopy for therapeutic purposes:					
Percutaneous transluminal coronary angioplasty	3	0.22	20.00	54.12	3.81
Other therapeutic interventional	2	0.14	20.00	34.52	2.43
Total	1219	100	—	1419.87	100.00

Computed tomography appears to be the highest contributor to the collective dose. While it amounts to only 9.6 % of the total frequency (117 per 1000 population), this radiological modality delivers 70.5 % of the dose (1.0 mSv per inhabitant). The CT frequency per 1000 population obtained in the work is 17 % higher than the 2008 figure in Switzerland (100) and is comparable with CT frequencies reported for 2012 in France (130)^(13)^ and in Germany (132)^(14)^.

The estimation of the uncertainties associated with these numbers is conditioned by the choice of the value used to extrapolate the national data from the authors’ sample. The authors processed, in the same way, the data corresponding to the extensive survey performed for 2008 to test their methodology. A very good agreement was found for the CT examinations (difference of 5 %). However, more discrepancies appeared for the conventional X-ray examinations (difference of 30 %). This is mainly due to the limited sample the authors got for the 2013 survey. Considering the importance of the CT contribution in terms of collective dose, the uncertainty associated with the average dose per inhabitant obtained in 2013 should be within 10 %.

**Figure 1.**
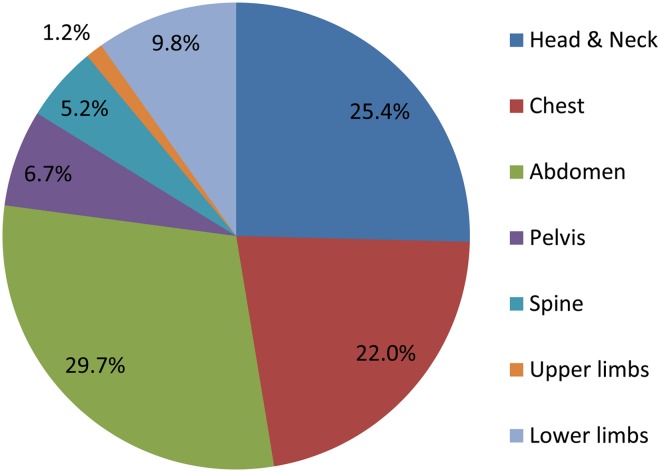
Distribution of CT examinations among the various regions of the body.

Figure 1 presents the distribution of CT examinations among the various regions of the body. The most frequent CT procedures are abdomen, head and neck, and chest.

**Figure 2.**
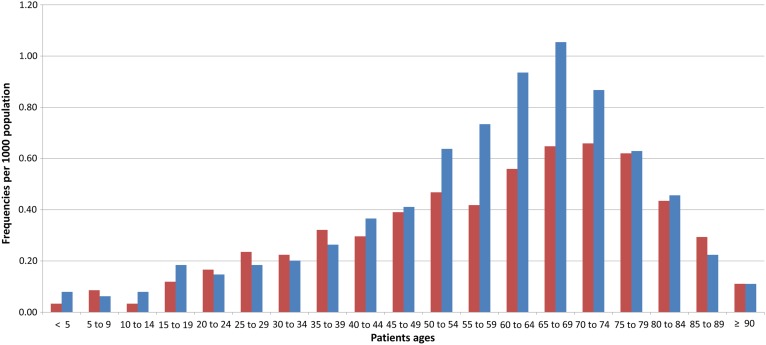
Age distribution of chest CT examinations. Blue (light gray) is for men and red (dark gray) is for women.

Figure 2 shows an example of age distribution obtained through the TARMED data. It concerns the CT of the chest, for which is observed a higher frequency for men than for women, except between 20 and 40 y.

Table 3 compares the dose data obtained in this survey with those reported in France and Germany for 2012. The average annual effective dose per inhabitant is compared by grouping modalities into three groups: X ray and fluoroscopy (including mammography and all uses of fluoroscopy), computed tomography and dental radiology. This excluded the contribution of nuclear medicine.

**Table 3.** Average annual effective dose per inhabitant (mSv) from various radiological modalities.

Year of the survey	This work 2013	France 2012^(13)^	Germany 2012^(14)^
X ray and fluoroscopy^a^	0.41	0.34	0.68
Computed tomography	1.00	1.14	1.13
Dental radiology	0.01	0.003	0.054
Rounded total^b^	1.40	1.50	1.80

^a^Group includes mammography and all uses of fluoroscopy.

^b^Excluding nuclear medicine.

## CONCLUSION

This survey indicated that the average annual number of examinations per inhabitant in Switzerland in 2013 was 1.2 and the associated annual effective dose was 1.4 mSv. The frequency of CT examinations has continued to increase from 2008 to 2013, leading to a 17 % increase in the average annual effective dose per inhabitant. Clinical and technical audits assessing the justification of CT procedures as well as optimising all related protocols are highly recommended in order to keep this dose-intensive radiological modality under control.
